# Conservation of the Red Kite *Milvus milvus* (Aves: Accipitriformes) Is Not Affected by the Establishment of a Broad Hybrid Zone with the Black Kite *Milvus migrans migrans* in Central Europe

**DOI:** 10.1371/journal.pone.0159202

**Published:** 2016-07-27

**Authors:** Petr Heneberg, Matej Dolinay, Hynek Matušík, Thomas Pfeiffer, Winfried Nachtigall, Jiří Bizos, Daniela Šimčíková, Ivan Literák

**Affiliations:** 1 Charles University in Prague, Third Faculty of Medicine, Prague, Czech Republic; 2 Masaryk University, Faculty of Science, Department of Botany and Zoology, Brno, Czech Republic; 3 Czech Society for Ornithology, Working Group for Protection and Research of Raptors and Owls, Březolupy, Czech Republic; 4 Rosenweg 1, Weimar, Germany; 5 Förderverein Sächsische Vogelschutzwarte Neschwitz e. V., Neschwitz, Germany; 6 University of Veterinary and Pharmaceutical Sciences Brno, Faculty of Veterinary Hygiene and Ecology, Department of Biology and Wildlife Diseases, Brno, Czech Republic; Sichuan University, CHINA

## Abstract

Among Accipitriformes sensu stricto, only a few species have been reported to form hybrid zones; these include the red kite *Milvus milvus* and black kite *Milvus migrans migrans*. *M*. *milvus* is endemic to the western Palearctic and has an estimated total population of 20–24,000 breeding pairs. The species was in decline until the 1970s due to persecution and has declined again since the 1990s due to ingestion of rodenticide-treated baits, illegal poisoning and changes in agricultural practices, particularly in its core range. Whereas F1 *M*. *milvus* × *M*. *migr*. *migrans* hybrid offspring have been found, F2 and F3 hybrids have only rarely been reported, with low nesting success rates of F1 hybrids and partial hybrid sterility likely playing a role. Here, we analyzed the mitochondrial (*CO1* and *CytB*) and nuclear (*Myc*) DNA loci of 184 *M*. *milvus*, 124 *M*. *migr*. *migrans* and 3 F1 hybrid individuals collected across central Europe. In agreement with previous studies, we found low heterozygosity in *M*. *milvus* regardless of locus. We found that populations of both examined species were characterized by a high gene flow within populations, with all of the major haplotypes distributed across the entire examined area. Few haplotypes displayed statistically significant aggregation in one region over another. We did not find mitochondrial DNA of one species in individuals with the plumage of the other species, except in F1 hybrids, which agrees with Haldane´s Rule. It remains to be investigated by genomic methods whether occasional gene flow occurs through the paternal line, as the examined *Myc* gene displayed only marginal divergence between *M*. *milvus* and *M*. *migr*. *migrans*. The central European population of *M*. *milvus* is clearly subject to free intraspecific gene flow, which has direct implications when considering the origin of individuals in *M*. *milvus* re-introduction programs.

## Introduction

Hybrid zones have recently received increasing attention, and several hybrid sterility genes have been identified, e.g., in house mouse *Mus musculus musculus* × *Mus musculus domesticus* hybrids [1–2] and in fruit flies *Drosophila pseudoobscura pseudoobscura* × *Drosophila pseudoobscura bogotana* [3]. In birds, the occurrence of hybrid zones is well documented [4]. Most observed bird hybrids have been found to be fertile, although their fertility was generally decreased compared to the parental types that produced them. The degree of fertility may also vary with age, sex, and the direction of the cross. With regard to sex, Haldane´s Rule applies [5], suggesting that when one sex is absent, rare, infertile, or inviable, it is virtually always the heterogametic sex, which is the female in birds. Thus, when two populations hybridize on an ongoing basis but the parental types of female-inherited mitochondrial DNA remain segregated, female hybrids are usually sterile [4]. Among Accipitriformes sensu stricto, only a few species have been reported to form hybrid zones. These include the red and black kites (*Milvus milvus* × *Milvus migrans migrans*) analyzed in this study; eagles (*Aquila clanga* × *Aquila pomarina*) as shown by Helbig et al. [6]; and marsh harriers (*Circus aeruginosus* × *Circus spilonotus*) as shown by Fefelov [7].

*M*. *milvus* is endemic to the western Palearctic, with an estimated total population of 20–24,000 breeding pairs [8]. Red kite populations declined until the 1970s due to persecution and have been declining again since the 1990s due to ingestion of rodenticide-treated baits, illegal poisoning and changes in agricultural practices, particularly in its core range, i.e., Spain, France and Germany. Populations in central and northern Europe are stable or increasing [8]. However, in central and northern Europe, its distribution overlaps with the closely related *M*. *migrans*. Black kites of the nominal subspecies *M*. *migr*. *migrans* (hereafter called black kites) breed in the Western Palearctic and Central Asia [9]. European *M*. *migr*. *migrans* comprise a relatively small proportion (~100,000 pairs) of the total population and suffers from poisoning, habitat degradation and wind energy development [8]. Hybridization between the two kite species has been documented repeatedly in Sweden and Germany, where the nesting ranges of red and black kites overlap, as well as in the Cape Verde Islands, which were invaded by the black kite relatively recently [4], with observations of hybrids across Europe and in winter quarters, including sub-Saharan Africa.

The increasing importance of red kite populations in the hybrid zone raises the question of whether there is gene flow between the declining red kite and the closely related black kite. We reviewed the known records of the nesting success of hybrid pairs and their offspring (Table 1). Whereas F1 hybrid offspring were produced without problems, F2 and F3 hybrids have rarely been reported. Nesting attempts of F1 hybrids were often unsuccessful, and hybrid appeared to be partially sterile. We thus hypothesized that partial hybrid sterility is responsible for the sympatric existence of both kite species despite the existence of hybrid zones and despite increasing numbers of red kites nesting within the hybrid zone in central Europe. As there have been few recorded direct observations, we sampled a large cohort of red and black kites collected within the hybrid zone and analyzed their mitochondrial and nuclear DNA to discern the presence or absence of hybrids other than F1s within sympatric populations of these two species.

**Table 1:** Review of past observations of hybrid *M*. *milvus* and *M*. *migr*. *migrans* individuals, and records of the nesting success of hybrid pairs and their offspring. The table was compiled based on [10–29].

Country	Nesting of hybrid pairs without sufficient documentation information available	Hybrid bird observed	Nest with F1 hybrid offspring	Nest with F2 hybrid offspring	Nest with F3 hybrid offspring
**BY**	1		♂*M*. *milv*. + ♀*M*. *migr*. →? pull., 1 fledged		
**CY**		1			
**CZ**	4		1		
			♂*M*. *milv*. + ♀*M*. *migr*. → 3 pull., 2 fledged		
			captive *M*. *milv*. + *M*. *migr*. → 2 nestings, Σ 3 pull. *		
**DE**	11		14	3, none successful	
			♂*M*. *milv*. + ♀*M*. *migr*. → 3 pull., 1 fledged	♂F1 + ♀*M*. *milv*. → many eggs with embryos, but no chicks	
			♂*M*. *migr*. + ♀*M*. *milv*. → 7 nestings, Σ 10 pull.	♂*M*. *migr*. + ♀F1 → 2 pull., none fledged	
			♂*M*. *migr*. + ♀*M*. *milv*. → 6 nestings, Σ 16 pull. **	♂*M*. *migr*. + ♀F1 → unsuccessful	
**DK**			1		
			♂*M*. *milv*. + ♀*M*. *migr*. → 3 pull., 2 fledged		
**ES**		1			
**FI**		1			
**GB**		1	1		
			*M*. *milv*. + *M*. *migr*. → 2 pull. *		
**GR**		1 ^†^			
**IT**	1	1			
**KE**		1			
**SE**			6	2	1
			♂*M*. *milv*. + ♀*M*. *migr*. → 6 nestings, Σ 17 pull.	♂*M*. *migr*. + ♀putative F1 → some fledged	♂F2 with *M*. *milv*. + ♀*M*. *milv*. → some fledged
				♂F1 + ♀*M*. *milv*. → 11 nestings, some fledged	
**SK**			2		
			♂*M*. *migr*. + ♀*M*. *milv*. → 2 nestings, Σ 5 pull.		

* Sex of parents was undisclosed.

** Male could be F1 hybrid.

^†^ Personal observation by I. Literák, H. Matušík and R. Petro (Lepenou, 23 and 24-Jan-2016).

## Material and Methods

### Study area and sampling

**Fig 1:**
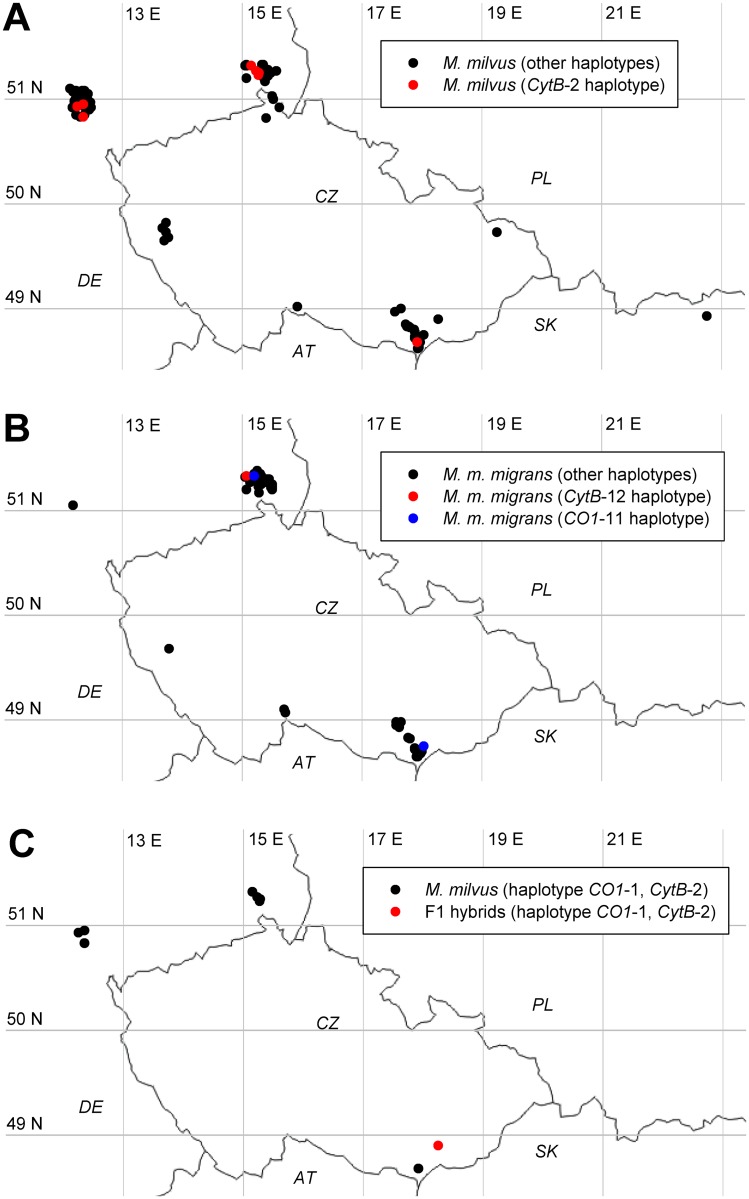
Location of study sites and haplotypes, the distribution of which was not uniform across the sampled region. Each dot represents one or more nests sampled at the indicated coordinates (with a precision to the nearest 0.01°N and 0.01°E. Samples were collected in Germany (172 individuals, 89 families), Czech Republic (136 individuals, 56 families) and Slovakia (3 individuals, 1 family). **(A)** *M*. *milvus* of *CytB*-2 haplotype (red dots) and other haplotypes (black dots). **(B)** *M*. *migr*. *migrans* of the *CytB*-12 haplotype (red dots), *CO1*-11 haplotype (blue dots) and other haplotypes (black dots). **(C)** Sampling site of the hybrid *M*. *milvus* × *M*. *migr*. *migrans* offspring (red dot) and of *M*. *milvus* individuals with the mitochondrial haplotype *CO1*-1, *CytB*-2, which was identical to that of the hybrids (black dots). Abbreviations of countries are indicated.

Samples were collected from 311 individuals representing 146 independent families (pulli or eggs within a single nest, including their parents if available; independent adults were each treated as a single family). Sampled individuals consisted primarily of pulli (n = 292), eggs or egg shells (n = 10), parents of the sampled offspring (n = 6), and independent adults found dead (n = 2) or alive (n = 1). Samples were collected in Germany (172 individuals, 89 families), the Czech Republic (136 individuals, 56 families) and Slovakia (3 individuals, 1 family) (Fig 1). According to plumage features, 184 individuals were *M*. *milvus*, 124 individuals were *M*. *migrans*, and three were their F1 hybrids. Samples were collected on 30-Mar-2013 (single adult found dead), between 22-Jun and 2-Jul-2013 (one hybrid nest and four control nests containing a total of nine juveniles used to establish analysis protocols), and between 23-May and 10-Jul-2014 (all other individuals).

From each live individual, we collected several down feathers and stored them in a freezer until analysis. Alternatively, we collected eggshells or eggs that did not hatch, down feathers of captured adult birds, fresh feathers of adults present below nests, and a liver tissue of an adult individual that was found dead. To avoid the possible inclusion of siblings and half-siblings, we sampled each territory only once, and 97% of individuals were sampled within a period of 48 days. Nestlings were sampled shortly before fledging when ringed and were identified based on plumage features and structural measurements. Care was taken to safely identify both adults attending each nest.

### DNA extraction and amplification

To extract DNA, feather samples were incubated at 65°C overnight in 2 ml of lysis buffer according to Smith et al. [30] with some modifications (10 mM Tris-HCl pH 7.5, 5 mM EDTA, 100 mM NaCl, 0.8 mg/ml proteinase K, 10 mg/ml sodium dodecyl sulfate). Following the complete lysis of the tissues, tubes were centrifuged for 1 min at 16,000×*g* and the supernatant was precipitated for 15 min at -20°C following addition of one volume of isopropanol. The precipitate was then pelleted by centrifugation for 15 min at 16,000×*g*, the pellet was washed using one volume of 75% ethanol and centrifuged for 5 min at 7,600×*g*. Pellets were then air-dried for 15 min at 60°C to remove residual ethanol and then dissolved in 100 μl of H_2_O for 15 min at 65°C. Two aliquots of obtained DNA were stored at -20°C. When contaminants were present, we purified the extractions using a NucleoSpin Tissue XS kit (Macherey-Nagel, Düren, Germany) according to the manufacturer’s instructions.

We amplified two mitochondrial (*CO1*, *CytB*) and two nuclear (*Myc*, *CHD*) DNA loci. The primers used for their amplification are specified in Table 2. The previously published *Myc* primers did not work well for *Milvus* spp. DNA samples. Therefore, we designed new *Myc* primers (MYC-R-04-mod, milv.Myc-in-fw and milv.Myc-in-rv) based on the conserved parts of the first few *M*. *milvus* and *M*. *migr*. *migrans* sequences generated in this study using Primer3 (http://frodo.wi.mit.edu/primer3, accessed on 13-Nov-2014). Resulting DNA amplicons were purified using USB Exo-SAP-IT (Affymetrix, Santa Clara, CA) and were subjected to bidirectional Sanger sequencing with an ABI 3130 DNA Analyzer (Applied Biosystems, Foster City, CA).

**Table 2:** Primers used for the amplification and sequencing of mitochondrial and nuclear DNA loci in *Milvus* spp.

Locus	Primer name	Sequence	Reference
*CO1*	BirdF1	TTCTCCAACCACAAAGACATTGGCAC	[31]
	CO1 R	ACTTCTGGGTGGCCAAAGAATCAGAA	[32]
*CytB*	L14996 cyt b	AACATCTCAATCTGATGAAACTTTGG	[33]
	L15828	GGAAGGTTATTGTGCGCTGT	[34]
*Myc*	MYC-F-02	TGAGTCTGGGAGCTTTATTG	[35]
	MYC-R-04-mod	GTGGGGCTTACTGTGCTCTTCT	This study
	milv.Myc-in-fw	TACTGCTGACAACCGAGGTTAAACTTTCC	This study
	milv.Myc-in-rv	AAACCCTTGGGAGATTCAGCCAAGG	This study
*CHD*	2550F	GTTACTGATTCGTCTACGAGA	[36]
	2718R	ATTGAAATGATCCAGTGCTTG	[36]

### Alignments and reconstruction of haplotype networks

Consensus DNA sequences were submitted to GenBank database under accession numbers KU640396-KU640408 (*CO1* haplotypes), KU670077-KU670091 (*CytB* haplotypes) and KU708627-KU708835 (*Myc* sequences) (S1 Table). The software Mixed Sequence Reader (available at <http://msr.cs.nthu.edu.tw/>) was used to analyze heterozygous *Myc* sequences when needed. Mitochondrial and nuclear sequences were trimmed relative to their shortest representative. For the *CO1* locus, the analyzed sequences corresponded to nucleotides 88–651 of KF946757. For the *CytB* locus, the analyzed sequences corresponded to nucleotides 149–641 of AY987312. For the *Myc* locus, the analyzed sequences corresponded to the partial intron B and exon 3 coding sequence, i.e., to nucleotides 36–387 of GU189490. To place the analyzed samples into a broader context, we performed GenBank BLASTn for each of the three loci using the following parameters: expected number of chance matches in a random model = 10; length of seed that initiates an alignment = 11; reward for matching bases = 1; penalty for mismatching bases = −3; gap cost—existence = 5, extension = 2. The search was limited to the genus *Milvus*. Previously obtained *CO1* sequences of *Milvus* spp. included in the analyses consisted of the unpublished one but available in the NCBI GenBank database (JN801326), and those published by Schindel et al. [37], Aliabadian et al. [38], Echi et al. [39], Saitoh et al. [40] and Gaikwad et al. [41]. Previously obtained *CytB* sequences of *Milvus* spp. included in the analyses consisted of those published by Seibold and Helbig [42–43], Johnson et al. [34] and Lerner and Mindell [44]. Previously obtained *Myc* sequences of *Milvus* spp. included in the analyses consisted of the unpublished sequences available in the NCBI GenBank database (GU189490 and GU189491). We reconstructed haplotype networks for the two mitochondrial loci separately, using TCS 1.21 [45]. Due to the high degree of heterozygosity in the nuclear *Myc* locus, we identified individual alleles for *M*. *milvus* only, whereas we analyzed *M*. *migr*. *migrans* samples only for the presence/absence of *M*. *milvus* alleles and the frequencies of individual polymorphisms for the rest of their sequences. The sexes of hybrid individuals were assessed based on their *CHD* locus sequences according to Fridolfsson and Ellegren [36].

### Ethics statement

Field work was only performed by licensed bird ringers who specialized in *Milvus* spp. ringing and who obtained all of the necessary permissions for the capture of pulli and collection of feathers as well as for access to sampling sites. The experimental design was approved by Departments of Environment of Regional Authorities of the South Moravian Region (permit No. JMK 18002/2014), Zlín Region (permit No. KUZL 9311/2014), Pilsen Region (permit No. ŽP/1307/14) and Ústí nad Labem Region (permit No. 85979/2014/KUUK). Preliminary data describing hybrid nesting of *M*. *milvus* and *M*. *migr*. *migrans* were published by the authors in the popular science journal Živa [10].

## Results

### *CO1* haplotype frequencies

**Fig 2:**
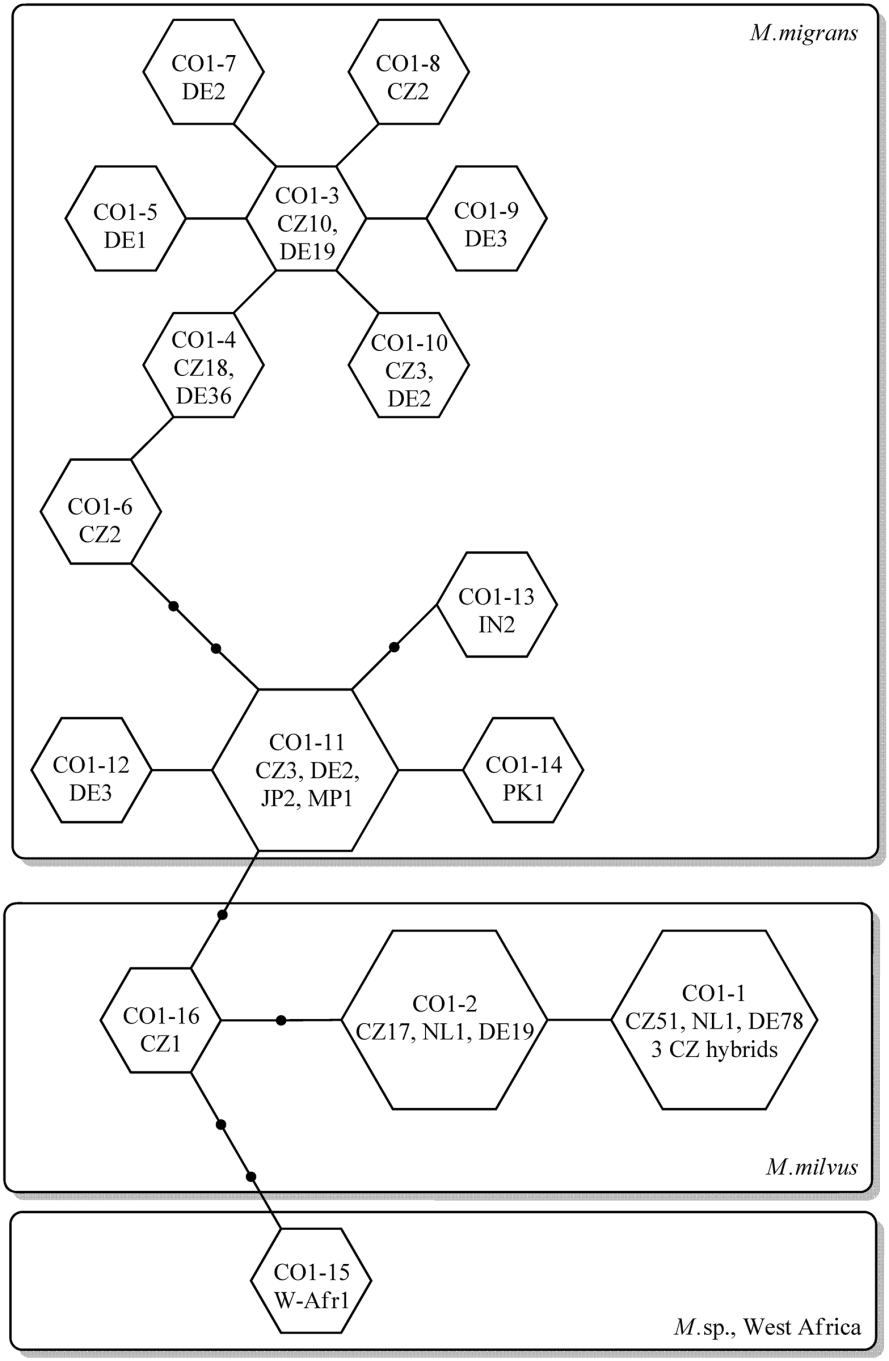
Statistical parsimony network among *Milvus milvus* × *Milvus migrans migrans* complex *CO1* locus haplotypes (numbered) constructed in TCS 1.21. Country-specific observed frequencies of each haplotype are indicated within circles. Lines indicate a single mutational step between haplotypes. Small black circles represent hypothesized unsampled or extinct haplotypes. Frames indicate species identities.

We identified 16 *CO1* haplotypes among our samples and GenBank sequences. These included ten haplotypes newly identified in this study, three haplotypes identified in previous studies but also present in our cohorts, and three haplotypes absent in the kites examined in this study. Whereas *M*. *migrans* was represented by 12 haplotypes, all previously examined *M*. *milvus* individuals only possessed three haplotypes, with a single haplotype associated with a kite from West Africa identified originally as *M*. *migrans* [39]. Note that Wink and Sauer-Gürth [46] suggested treating sub-Saharan populations of kites as a distinct species (*Milvus aegyptius*) due to differences in their *CytB* sequences, which is also supported by morphology (yellow color of the beak). Aside from the latter West African individual, all *M*. *migrans* and *M*. *milvus* haplotypes formed two distinct species-specific haplotype networks under the statistical parsimony 95% confidence interval (Fig 2; Tables 3 and 4).

**Table 3:** Variable sites within the analyzed *CO1* haplotypes of *M*. *milvus* and *M*. *migr*. *migrans*.

		Position																				
Haplotype	Species	75	87	114	153	204	207	252	262	282	291	294	300	310	342	390	447	486	487	528	543	546
*CO1*-1	*M*. *milvus*	A	T	T	G	C	C	C	G	G	A	T	C	T	G	T	A	G	T	T	T	C
*CO1*-2	*M*. *milvus*	.	.	.	.	.	.	.	.	.	.	.	.	.	A	.	.	.	.	.	.	.
*CO1*-3	*M*. *migr*. *migrans*	.	C	.	A	.	.	T	.	A	G	.	.	.	A	.	.	.	C	A	.	T
*CO1*-4	*M*. *migr*. *migrans*	G	C	.	A	.	.	T	.	A	G	.	.	.	A	.	.	.	C	A	.	T
*CO1*-5	*M*. *migr*. *migrans*	.	C	.	A	.	.	T	.	A	G	.	.	.	A	.	C	.	C	A	.	T
*CO1*-6	*M*. *migr*. *migrans*	G	C	.	A	.	.	.	.	A	G	.	.	.	A	.	.	.	C	A	.	T
*CO1*-7	*M*. *migr*. *migrans*	.	C	.	A	.	.	T	.	A	G	.	.	C	A	.	.	.	C	A	.	T
*CO1*-8	*M*. *migr*. *migrans*	.	C	.	A	.	.	T	.	A	G	.	.	.	A	C	.	.	C	A	.	T
*CO1*-9	*M*. *migr*. *migrans*	.	C	.	A	.	.	T	.	A	G	.	T	.	A	.	.	.	C	A	.	T
*CO1*-10	*M*. *migr*. *migrans*	.	C	.	A	T	.	T	.	A	G	.	.	.	A	.	.	.	C	A	.	T
*CO1*-11	*M*. *migr*. *migrans*	G	C	.	.	.	.	.	.	.	G	.	.	.	A	.	.	.	.	.	.	T
*CO1*-12	*M*. *migr*. *migrans*	G	C	.	.	.	.	.	.	.	G	C	.	.	A	.	.	.	.	.	.	T
*CO1*-13	*M*. *migr*. *migrans*	G	C	.	.	.	T	.	.	.	G	.	.	.	A	.	.	A	.	.	.	T
*CO1*-14	*M*. *migr*. *migrans*	G	C	.	.	.	.	.	A	.	G	.	.	.	A	.	.	.	.	.	.	T
*CO1*-15	*M*. *migr*. *migrans*	.	.	C	.	.	.	.	.	A	G	.	.	.	A	.	.	.	.	.	C	T
*CO1*-16	*M*. *milvus*	.	.	.	.	.	.	.	.	.	G	.	.	.	A	.	.	.	.	.	.	T

**Table 4:** Variable sites within the analyzed *CytB* haplotypes of *M*. *milvus* and *M*. *migr*. *migrans*.

		Position																															
Haplotype	Species	3	15	37	54	66	84	87	99	109	144	147	177	183	222	235	237	267	270	294	297	331	363	375	411	435	436	437	444	456	468	477	489
CytB-1	*M*. *milvus*	C	C	C	A	T	A	C	A	T	T	A	G	A	A	G	A	C	T	A	C	T	C	C	C	C	A	T	A	G	A	A	A
CytB-2	*M*. *milvus*	.	.	.	G	.	.	.	.	.	.	.	.	.	.	.	.	.	.	.	.	.	.	.	.	.	.	.	.	.	.	.	.
CytB-3	*M*. *migr*. *migrans*	.	A	.	.	C	G	.	.	.	C	.	.	.	.	.	G	.	.	.	.	.	T	.	.	T	.	.	.	A	.	.	G
CytB-4	*M*. *migr*. *migrans*	.	A	.	.	C	G	.	.	.	.	.	.	.	.	.	G	.	.	.	.	.	T	.	.	T	.	.	.	A	.	.	G
CytB-5	*M*. *migr*. *migrans*	.	A	.	.	C	G	.	.	.	.	.	.	.	.	.	G	.	C	.	.	.	T	.	.	T	.	.	.	A	.	.	G
CytB-6	*M*. *migr*. *migrans*	.	.	.	.	C	.	.	.	.	.	.	.	C	.	.	G	.	.	.	.	.	T	.	.	T	.	.	.	A	.	.	G
CytB-7	*M*. *milvus*	.	.	.	.	.	.	.	.	.	.	.	.	.	.	.	.	.	.	.	.	.	.	.	A	.	.	.	.	.	.	.	.
CytB-8	*M*. *milvus*	T	.	.	.	.	.	.	.	.	.	.	.	.	.	.	.	.	.	.	.	.	.	.	.	.	.	.	.	.	.	.	.
CytB-9	*M*. *milvus*	.	.	.	G	.	.	.	.	.	.	.	.	.	.	A	.	.	.	.	.	.	.	.	.	.	.	.	.	.	.	.	.
CytB-10	*M*. *migr*. *migrans*	.	A	.	.	C	G	.	.	.	.	.	.	.	.	.	G	.	.	.	.	.	T	.	.	T	.	.	.	A	.	.	.
CytB-11	*M*. *migr*. *migrans*	.	A	.	.	C	G	.	.	.	.	.	.	.	.	.	G	T	.	.	.	.	T	.	.	T	.	.	.	A	.	.	G
CytB-12	*M*. *migr*. *migrans*	.	A	.	.	C	G	.	.	.	.	.	.	.	.	.	G	T	.	.	.	C	T	.	.	T	.	.	.	A	.	.	G
CytB-13	*M*. *migr*. *migrans*	.	A	.	.	C	G	.	.	.	.	.	.	.	.	.	G	.	.	.	.	.	T	.	.	T	.	C	.	A	.	.	G
CytB-14	*M*. *migr*. *migrans*	.	.	.	.	C	.	.	.	.	.	.	.	.	.	.	G	.	.	.	.	.	T	.	.	T	.	.	.	A	.	.	G
CytB-15	*M*. *migr*. *migrans*	.	A	.	.	C	G	A	.	C	.	.	A	.	.	.	G	.	.	.	.	.	T	.	.	T	.	.	.	A	.	.	G
CytB-16	*M*. *migr*. *migrans*	.	A	.	.	C	G	.	.	.	.	G	.	.	.	.	G	.	.	.	.	.	T	.	.	T	.	.	.	A	.	.	G
CytB-17	*M*. *migr*. *migrans*	.	A	.	.	C	G	.	.	.	.	.	.	.	.	.	.	.	.	.	.	.	T	.	.	T	.	.	.	A	.	.	G
CytB-18	*M*. *migr*. *lineatus*	.	.	.	.	C	.	.	.	.	.	.	.	.	G	.	G	.	.	.	.	.	T	.	.	T	.	.	.	A	G	.	G
CytB-19	*M*. *migr*. *govinda*	.	.	.	.	C	.	.	.	.	.	.	.	.	.	.	G	.	.	.	.	.	T	.	.	T	.	.	.	A	G	.	G
CytB-20	*M*. *migr*. *affinis*	.	.	.	.	C	.	.	.	.	.	.	.	.	.	.	G	.	.	C	.	.	T	.	.	T	.	.	G	A	.	.	G
CytB-21	*M*. *migr*. *parasiticus*	.	.	.	.	.	.	.	T	.	.	.	.	.	.	.	.	.	.	.	.	.	T	.	.	T	G	.	.	A	.	.	G
CytB-22	*M*. *migr*. *parasiticus*	.	.	T	.	.	.	T	.	C	.	.	.	.	.	.	G	.	.	.	T	.	T	T	.	.	.	.	.	A	.	G	.

Haplotypes *CO1*-4, *CO1*-3 and *CO1*-11 accounted for 81% of all of the *M*. *migrans* individuals. Haplotype *CO1*-3 differed from *CO1*-4, -5, -7, -8, -9 and -10 by a single nucleotide change, and from *CO1*-6 by two substitutions. All of the latter haplotypes were specific to the examined central European region, and were not identified among haplotypes retrieved from the NCBI Nucleotide database. In contrast, the third most common haplotype, *CO1*-11, was relatively rare in central Europe (three Czech individuals and two German individuals), but was also present in two Japanese individuals and in a kite from the North Mariana Islands. Individuals from Pakistan and India also possessed haplotypes that were highly similar to this one, with only one or two nucleotide substitutions. Only two nucleotide substitutions separated this haplotype from the closest *M*. *milvus* haplotype.

In *M*. *milvus*, the haplotype *CO1*-1 accounted for 130 (77%) of all *M*. *milvus* individuals and also for the three F1 *M*. *milvus* × *migrans* hybrids examined. The second most common haplotype, *CO1*-2, accounted for 22% of *M*. *milvus* individuals tested. Both of these haplotypes were reported previously from the Netherlands. Additionally, we identified a single individual with haplotype *CO1*-16, which was the closest haplotype to those identified in *M*. *migrans* (Fig 2).

### *CytB* haplotype frequencies

**Fig 3:**
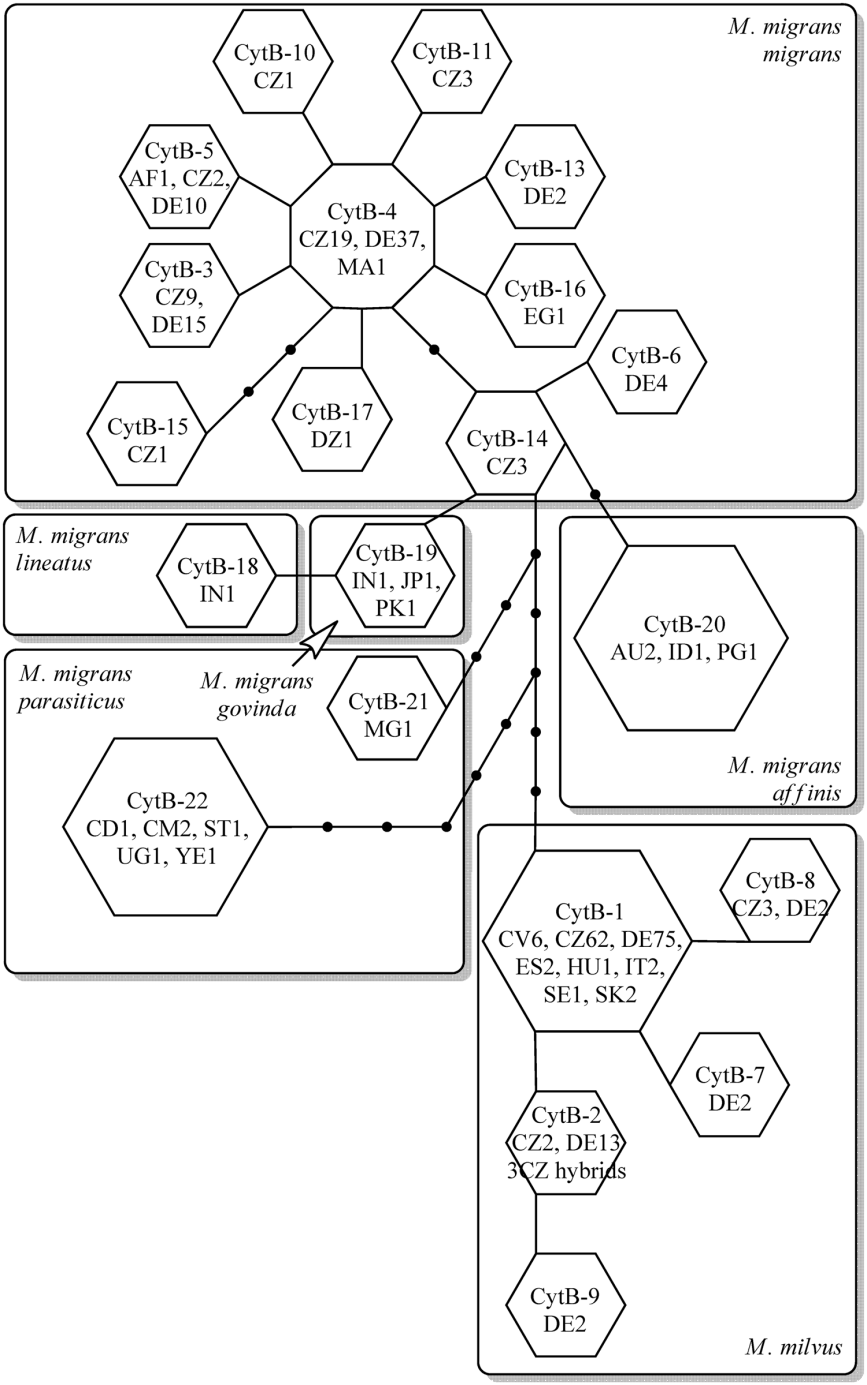
Statistical parsimony network among *Milvus milvus* × *Milvus migrans migrans* complex *CytB* locus haplotypes (numbered) constructed in TCS 1.21. Country-specific observed frequencies of each haplotype are indicated within the circles. Lines indicate a single mutational step between haplotypes. Small black circles represent hypothesized unsampled or extinct haplotypes. Frames indicate species or subspecies identities.

We identified 22 *CytB* haplotypes among the kites tested in this study and GenBank sequences. These included 11 haplotypes newly identified in this study, three haplotypes identified in previous studies but also present in our cohorts, and eight haplotypes absent in the kites examined in this study. Whereas *M*. *migrans* was represented by 17 haplotypes, all previously examined *M*. *milvus* individuals only possessed five haplotypes. The *CytB* locus distinguished between haplotypes of the *M*. *migrans* subspecies, i.e., *M*. *migr*. *migrans*, *Milvus migrans affinis*, *Milvus migrans govinda*, *Milvus migrans lineatus* and *Milvus migrans parasiticus*. Note that t he black-eared kite *M*. *migr*. *lineatus* was described as a distinct phylogenetic lineage based on genetic (*CytB*) and morphological differences from *M*. *migr*. *migrans* according to Scheider et al. [47]. The species rank was also assigned to *M*. *migr*. *lineatus* by Sibley and Monroe [48]. All haplotypes formed distinct subspecies-specific (*M*. *migrans*) or species-specific (*M*. *milvus*) haplotype networks under the statistical parsimony 95% confidence interval (Fig 3).

The haplotypes *CytB*-4, *CytB*-3 and *CytB*-12 accounted for 86% of all central European *M*. *migrans migrans* individuals. The haplotype *CytB*-4 differed from *CytB*-3, -5, -10, -11, -13, -16 and -17 by a single nucleotide change, from *CytB*-14 by two substitutions, and from *CytB*-15 and -6 by three substitutions. These haplotypes were found in multiple regions outside of central Europe. The core haplotype, *CytB*-4, was previously reported in Morocco. Another common haplotype, *CytB*-5, was previously reported in *M*. *migr*. *migrans* from Afghanistan. Seven haplotypes were only found in central Europe (including the common haplotype *CytB*-3). Two haplotypes were absent in central Europe, including *CytB*-16 (found in *M*. *migr*. *migrans* from Egypt) and *CytB*-17 (found in *M*. *migr*. *migrans* from Algeria). Five haplotypes represented other *M*. *migrans* subspecies. All were more distant from the core *M*. *migr*. *migrans* haplotype *CytB*-4 than other *M*. *migr*. *migrans* haplotypes, suggesting that *CytB* supports the validity of these independent subspecies.

In *M*. *milvus*, the haplotype *CytB*-1 accounted for 151 (86%) of all *M*. *milvus* individuals. This was the only haplotype previously reported, and was identified repeatedly in *M*. *milvus* from multiple central European countries, Estonia, Italy and Cabo Verde. All other haplotypes were newly identified in this study. The second most common haplotype, *CytB*-2, accounted for 9% of *M*. *milvus* individuals, including the three F1 *M*. *milvus* × *M*. *migr*. *migrans* hybrids examined (Fig 1C). Haplotypes *CytB*-1 and *CytB*-2 differed by a single nucleotide substitution. We also identified three additional haplotypes, each differing by only a single nucleotide substitution from either *CytB*-1 or *CytB*-2, and each represented by two to five individuals (Fig 3).

### Polymorphisms in the nuclear *Myc* gene

The reference sequences (*M*. *milvus* GU189490 nt. 36–387, and *M*. *migrans* GU189491 nt. 36–384), based on which we selected this region among other nuclear genes with known *M*. *milvus* and *M*. *migrans* sequence, differed by three homozygous nucleotide substitutions and a single triplet codon deletion. In addition, the reference *M*. *migrans* sequence contained four heterozygous sites. Based on the analysis of 120 *M*. *milvus* sequences, 68 *M*. *migr*. *migrans* sequences and three sequences of their F1 hybrids, we identified 25 polymorphic sites within the examined partial sequence of *Myc* gene (Table 5).

**Table 5:** Polymorphisms in the nuclear *Myc* gene. As reference sequences, we used *M*. *milvus* GU189490 nt. 36–387, and *M*. *migrans* GU189491 nt. 36–384.

Species	*M*. *milvus*			*M*. *migrans*			1^st^ generation hybrids		
Σ n	120			68			3		
Polymorphism	Number of homozygotes for the major allele	Number of heterozygotes	Number of homozygotes for the minor allele	Number of homozygotes for the major allele	Number of heterozygotes	Number of homozygotes for the minor allele	Number of homozygotes for the major allele	Number of heterozygotes	Number of homozygotes for the minor allele
6C>T	120	0	0	67	0	1	3	0	0
8C>T	120	0	0	66	1	1	3	0	0
9T>A	120	0	0	67	1	0	3	0	0
14C>T	120	0	0	14	15*	39	2	1	0
17T>C	120	0	0	67	1	0	3	0	0
29C>T	120	0	0	68	0*	0	3	0	0
31C>T	120	0	0	67	1	0	3	0	0
34C>T	120	0	0	64	1	3	3	0	0
34C>G	120	0	0	63	1	4	3	0	0
56G>A	120	0	0	25	11	32*	2	1	0
63C>T	120	0	0	46	14	8	1	2	0
83G>A	120	0	0	65	2*	1	3	0	0
87G>A	120	0	0	67	1	0	3	0	0
102A>G	120	0	0	65	3	0	3	0	0
113C>A	120	0	0	67	1	0	3	0	0
115T>C	120	0	0	67	0	1	3	0	0
127C>T	88	20	12	28	16*	24	2	1	0
136G>A	90	19	11	23	20	25*	2	1	0
142G>A	90	19	11	3	3	62*	0	3	0
149G>A	90	19	11	3	4	61*	0	3	0
176C>T	120	0	0	52	9	7	3	0	0
206A>G	120	0	0	49	11	8	3	0	0
219C>T	120	0	0	67	1	0	3	0	0
262T>G	120	0	0	67	0	1	3	0	0
284-6delGTT	120	0	0	41	20	7*	3	0	0

* Indicates the reference sequence when it differs from the most frequent haplotype.

In *M*. *migrans*, all 25 sites were polymorphic and some positions were highly polymorphic. The following substitutions characteristic for *M*. *milvus* were also present in *M*. *migrans* as polymorphisms with frequencies equal or higher than 10%: 14C>T (f_T_ = 68%), 56G>A (f_A_ = 55%), 63C>T (f_T_ = 22%), 127C>T (f_T_ = 47%), 136G>A (f_A_ = 51%), 142G>A (f_A_ = 93%), 149G>A (f_A_ = 93%), 176 C>T (f_T_ = 17%), 206A>G (f_G_ = 27%), and 284-6delGGT (f_del_ = 25%). Thus, all of the common polymorphisms were transitions. The haplotypes identified in two individuals (3LF-2163 and 3LF-2169) were identical with haplotypes found in *M*. *milvus*; all of the other haplotypes differed by two or more nucleotide substitutions or by the codon deletion absent in *M*. *milvus*. The two putative hybrids originated from two independent nests (near Teicha and Commerau, both in Upper Lusatia, Germany). Sample 3LF-2169 was heterozygous for the *M*. *milvus Myc* haplotype, whereas sample 3LF-2163 was homozygous for the *M*. *milvus Myc* haplotype. The *Myc* gene was also amplified from their siblings (one per nest), but these siblings did not carry the *Myc* alleles that are shared with *M*. *milvus*. The *CO1* and *CytB* loci were negative for *M*. *milvus* haplotypes in all pulli examined from these two nests. We cannot exclude that the alleles found in 3LF-2163 and 3LF-2169 simply represent low-frequency genotypes of *M*. *migrans* or that they originated by random mutations. However, at least two mutations (or a triplet insertion) would be needed to generate such alleles from those solely associated with *M*. *migrans*.

In *M*. *milvus*, only four sites were polymorphic, and all others were identical to the reference genotype. Polymorphic nucleotides included the transitions 127C>T, 136G>A, 142G>A and 149G>A; we did not detect any transversions. These four sites were also polymorphic in *M*. *migr*. *migrans*, and thus cannot be used for species discrimination. We identified three combinations of these alleles, the highly prevalent CGGG (82% of alleles), the less prevalent TAAA (17% of alleles), and the rare TGGG (1% of alleles). The frequency of the most common combination CGGG reached 7% in *M*. *migr*. *migrans*. Importantly, all other sites in which *M*. *migrans* was highly heterozygous (e.g., 14C>T, 56G>A, 63C>T and 284-6delGTT) were not heterozygous in any single *M*. *milvus* individual. Thus, analysis of *Myc* sequences indicated the absence of F1+n hybrids (when n≥2) among the examined 120 *M*. *milvus* individuals.

The three F1 hybrids examined were heterozygous for 14C>T, 56G>A (one individual each), 63C>T (two individual), 127C>T, 136G>A (one individual each), 142G>A, and 149G>A (all three individuals) (Table 5).

### Conservation genetics of kites

**Fig 4:**
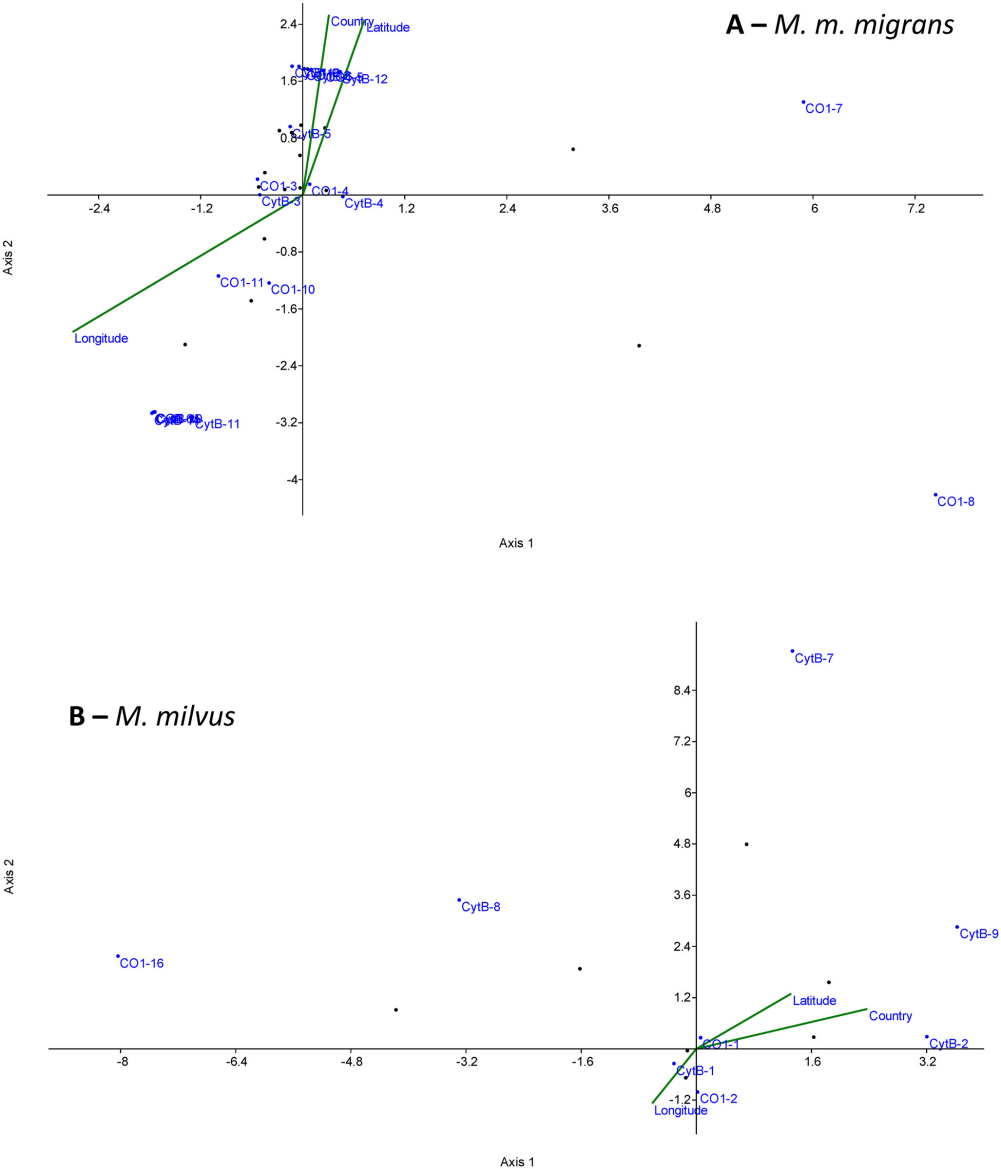
Correspondence analysis (Benzecri scaling) of *CO1* (A) and *CytB* (B) haplotypes according to [49]. We used a matrix of samples with assigned environmental variables (latitude, longitude, and country). The ordination axes are linear combinations of the environmental variables according to the Eigen analysis algorithm, with ordinations given as the site scores, and environmental variables are plotted as correlations with the site scores. Type 1 scaling according to [49] was used. The following data transformations were used: Country: 1 = SK, 2 = CZ, 3 = DE, 4 = other. The resulting factor scores of correspondence analyses are indicated in the text.

Mitochondrial haplotypes differed not only between species, but also in relation to the longitude and latitude of nest sites and some were restricted to certain countries (one-way ANOVA *p*<<0.001; for post-test outcomes see Table 6). We therefore performed canonical correlation analysis of *CO1* and *CytB* haplotypes for central European *Milvus* individuals for which both of these loci were successfully sequenced (n = 158 *M*. *milvus*, n = 102 *M*. *migrans*) (Fig 4).

**Table 6:** Outcomes of one-way ANOVA and post-tests analyzing the distribution of mitochondrial haplotypes according to longitude and latitude of nest sites and distribution in specific countries. The following data transformations were used: Country: 1 = SK, 2 = CZ, 3 = DE, 4 = other. Species: 1 = *M*. *milvus*, 2 = hybrid *M*. *milvus* × *M*. *migr*. *migrans*, 3 = *M*. *migr*. *migrans*, 4 = other subspecies of *M*. *migrans*, 5 = West-African *Milvus* sample.

	Sum of sqrs	df	Mean square	F	p(same)	
Between groups:	566102	5	113220	19040.0	0.00	
Within groups:	11250.1	1892	5.95			
Total:	577352	1897				
omega^2:	0.9805					
Levene's test for homogeneity of variance, based on means: p(same) =					1.87E-71	
Based on medians: p(same) =					5.41E-44	
Welch F test in the case of unequal variances:					F = 9.519E04, df = 815.8, p = 0	
Tukey's pairwise comparisons: Q below diagonal, p(same) above diagonal:						
	*CO1*	*CytB*	Country	Species	Latitude	Longitude
*CO1*		7.6E-04	0.8101	0.00	0.00	2.0E-05
*CytB*	5.7		2.2E-05	0.00	0.00	2.0E-05
Country	1.8	7.5		0.00	0.00	2.0E-05
Species	7.1	12.8	5.4		0.00	2.0E-05
Latitude	344.3	338.6	346.1	351.4		2.0E-05
Longitude	83.7	77.9	85.4	90.8	260.7	

In *M*. *migrans*, axis 1 explained 70.5% of the variance (eigenvalue 0.41), and axis 2 explained 29.5% of the variance (eigenvalue 0.17). The dominant haplotypes *CO1*-4, *CO1*-3, *CytB*-4 and *CytB*-3 were insensitive to geographic variation. In contrast, the dominant haplotype *CO1*-11 was associated with higher longitudes and lower latitudes. The dominant haplotype *CytB*-12 was associated with higher latitudes, particularly with German sampling sites (Figs 4A and 1B). Minor haplotypes were mostly associated with single countries, and only *CO1*-10 and *CytB*-5 displayed a mixed pattern.

In *M*. *milvus*, axis 1 explained 84.9% of the variance (eigenvalue 0.05), and axis 2 explained 15.1% of the variance (eigenvalue 0.01). The dominant haplotypes *CO1*-1, *CO1*-2 and *CytB*-1 were insensitive to geographic variation. The *CytB*-2 haplotype (which was also found in the F1 hybrids) was associated with higher latitudes and Germany (Figs 4B, 1A and 1C). Other haplotypes were found in few samples.

### Hybridization in kites

**Fig 5:**
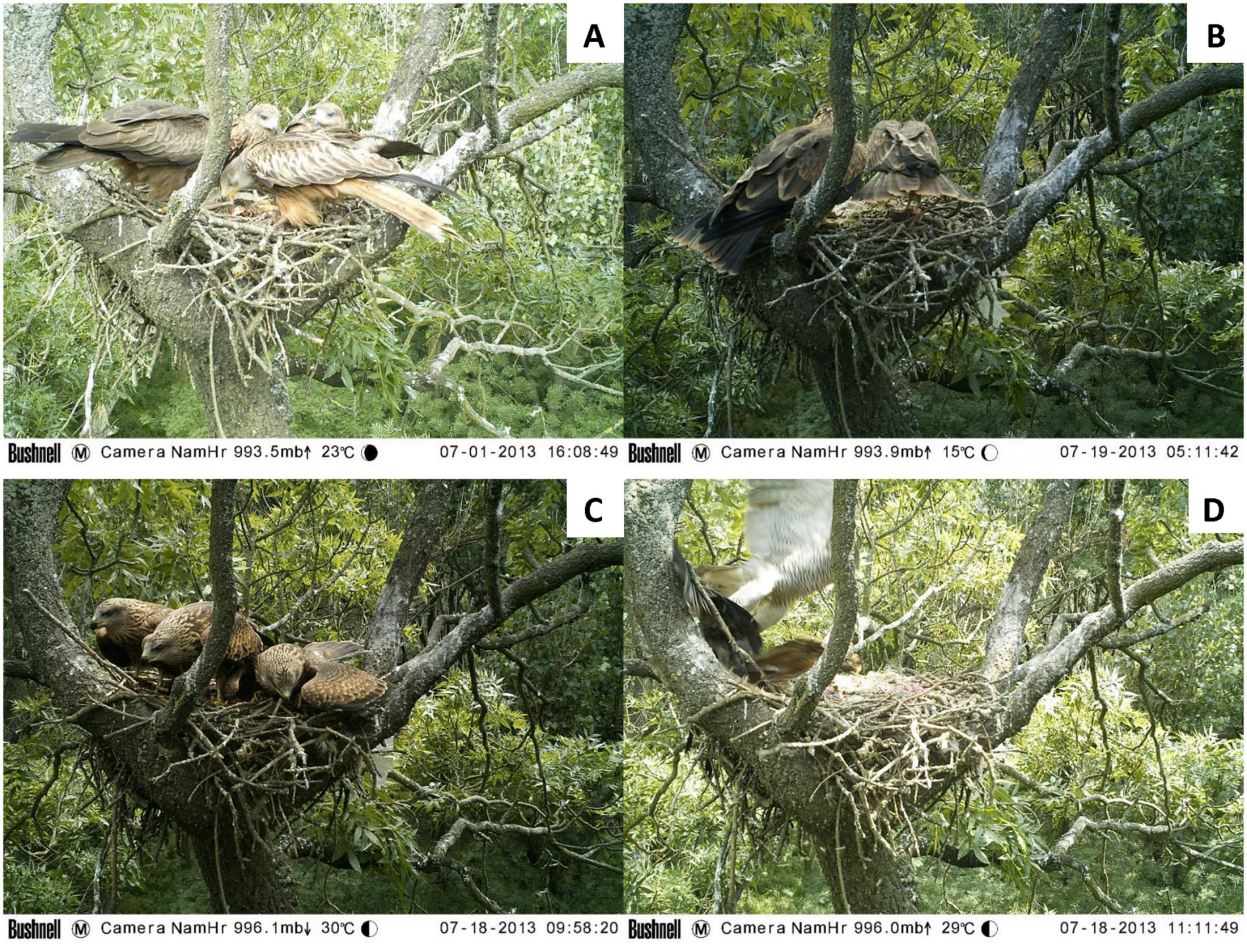
Successful nesting of a hybrid pair of kites consisting of male *M*. *migr*. *migrans* and female *M*. *milvus* in 2013 in SE Czech Republic. **(A)** Male *m*. *migrans* and female *M*. *milvus* present together at a nest. **(B)** Female and one of their hybrid offspring. **(C)** Hybrid offspring. **(D)** Hybrid kite pulli are attacked successfully by *Accipiter gentilis*.

We observed and documented a successful nesting of a hybrid pair of kites consisting of a male *M*. *migr*. *migrans* and a female *M*. *milvus* in 2013 (Fig 5A). The nest, located in SE Czech Republic, was occupied by a pair of *M*. *migr*. *migrans* in 2012, but in the next year a female *M*. *milvus* and a male *M*. *migr*. *migrans* were observed there. The nest was located in an alluvial forest near the Morava river, and was first visited on 14 April 2013. After one month a *M*. *milvus* female was sitting on the nest (13 May 2013). The nest was full of textile pieces and plastic (this material is used more frequently in nests of *M*. *milvus* than *M*. *migr*. *migrans*; H. Matušík, pers. obs.). A male black kite was observed nearby on that day. Both individuals were observed on 7 June 2013 sitting together near the nest. We then confirmed three young birds that were approximately ten days old, which did not fully correspond with either *M*. *milvus* or *M*. *migr*. *migrans* offspring and with particular individuals that differed from one another (Fig 5B and 5C). Analysis of the *CDH* locus revealed that all three pulli were males (data not shown).

The presence of both parents together in the nest was documented very rarely. The nest was regularly visited by both parents, but the frequency of visits by the female was higher. A Bushnell phototrap revealed the presence of another *M*. *milvus* individual on the nest (22 June 2013). This adult had clearly different plumage than the female.

On 18 July 2013, a few days before fledging, they were attacked by an *Accipiter gentilis* (Fig 5D). One individual was captured and the leftovers (especially feathers) were found on 30 July 2013 approximately 30 m from the nest tree. The two remaining kites relocated near the nest for the next few days and occasionally returned back to the nest for food. The last time the presence of a young or adult individual was recorded was on 24 July 2013, and monitoring was ceased on 30 July 2013.

**Fig 6:**
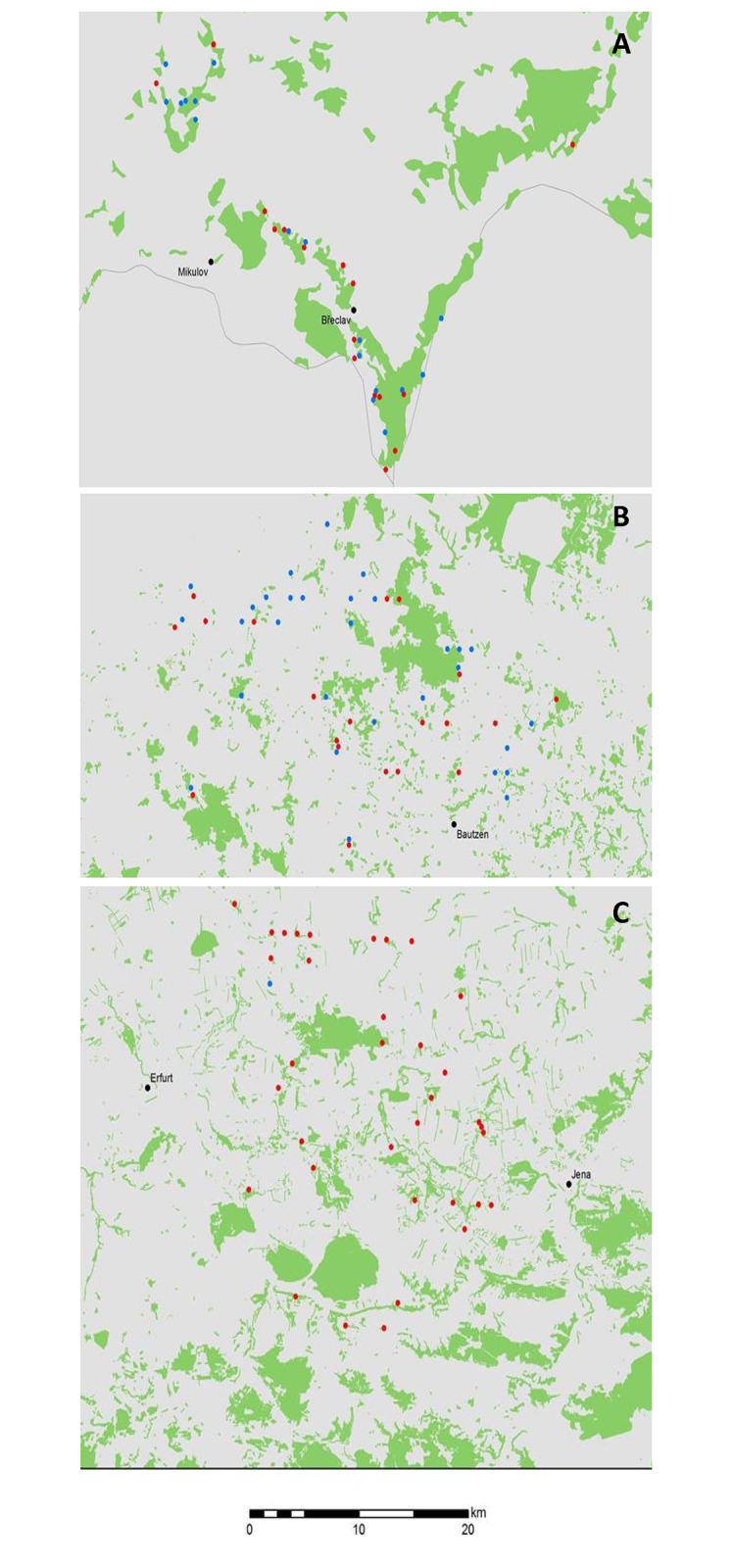
Nest sites of both *M*. *milvus* and *M*. *migr*. *migrans* co-localized with the distribution of forest patches. The nests of both species were localized to forest edges and to small forest fragments (including hedgerows and oak alleys at dams). Both avoided large forest complexes, and the nests of both species were frequently close to one another. The species occupied identical habitat across central Europe despite the landscape differing substantially between the agricultural lowlands of South Moravia **(A)**, heath and pond landscape of Saxonian Upper Lusitania **(B)** and hilly landscape of Thuryngia with many forest complexes and linear forest fragments **(C)**. Red dots denote *M*. *milvus* sampling nest sites, blue dots denote *M*. *migr*. *migrans* sampling nest sites, black dots show major cities, and green areas show the extent of forests.

In Germany, Czech Republic and Slovakia, nests of both species were localized to forest edges and to small forest fragments (including hedgerows and oak alleys at dams). Both avoided large forest complexes, and the nests of both species were frequently close to one another (Fig 6). Despite this proximity of nest sites, and despite finding a nest with F1 hybrids, we did not identify any pairs with a F1 hybrid as a parent. In agreement with Haldane´s Rule, we did not find any F1+n hybrids (where n≥2) where the heterogametic sex would be responsible for hybridization (i.e., no F1+n individuals where the maternal lineage would include an individual of the other species, such as *M*. *milvus* by plumage with the mitochondria of *M*. *migr*. *migrans*, or *M*. *migr*. *migrans* by plumage with the mitochondria of *M*. *milvus*). In contrast, we identified two individuals with *Myc* genotypes suggesting past hybridization events.

## Discussion

The phenotypes of two species of the genus *Milvus* in Europe are clearly distinguished by their characteristic tail forks, plumage color and some aspects of breeding and migration behavior [11]. The genetic distance between *M*. *milvus* and *M*. *migr*. *migrans* populations in Europe is shaped by their recent divergence during one of the last glaciations of the Pleistocene [50]. Previously, allozymes were used to distinguish between multiple individuals of *M*. *milvus* and *M*. *migr*. *migrans* [51]. Schreiber et al. found that the species were close to one another, reporting a Nei's interspecies genetic distance of D = 0.009, with 15.4% of the variability of four polymorphic enzymes being attributed to the differentiation between kite species and 84.6% contributed by within species polymorphism. Allozymes permitted the identification of just 78% of individuals of *M*. *milvus* and only a striking 5.4% of *M*. *migr*. *migrans* individuals [51]. Later, several hypervariable loci were sequenced in both kite species. These included *CO1*, *CytB*, *ND2*, *Myc*, and others. Particularly Johnson et al. [34] analyzed numerous *Milvus* individuals while investigating the validity of *M*. *milvus fasciicauda*, the extinct Cape Verde kite. They sequenced several *M*. *milvus* and *M*. *migrans* individuals of European origin for their *CytB* and *ND2* loci, showing that they display considerable sequence variability with *M*. *milvus* and *M*. *migr*. *migrans* forming separate clusters, but with *M*. *milvus* falling out as an ingroup of *M*. *migrans* [34]. In the same year, Roques and Negro [50] published an analysis of the mitochondrial control region in *M*. *milvus*, concluding that there is very low genetic diversity in the mitochondrial control region of *M*. *milvus*, ranging from zero in Majorca to 0.0062 in central Europe, with a mean sequence divergence of only 0.75%. The core areas of the *M*. *milvus* distribution (areas with the highest heterozygosity) were identified as central Europe and central Spain, whereas southern Spain and island locations displayed the lowest heterozygosity. Surprisingly, the authors assumed that the observed low heterozygosity was caused by successive bottlenecks and small population sizes [50]. However, such effects should be more prominent in central Europe, which was subject to repeated glaciation events, whereas the southern parts of Europe may have provided better living conditions at those times. Thus, this indicates that the low DNA heterozygosity in *M*. *milvus* suggests a recent speciation event rather than a bottleneck effect. Aside from these two studies, several other researchers have analyzed various DNA loci in kites. Among them were Oatley et al. [52], who sequenced *M*. *migrans* for ND1, including the flanking tRNA regions, and also three nuclear introns consisting of myoglobin intron-2, beta fibrinogen intron-5 and TGFß2 intron-5. Burri et al. [53] reported MHC class IIB genes of *M*. *milvus*. Saitoh et al. [40] and Gaikwad et al. [41] provided *CO1* sequences of *M*. *migrans*, and Aliabadian et al. [38] provided the *CO1* sequence of *M*. *milvus*. Matsuki [54] patented the ND6 sequence of *M*. *migrans*.

In this study, we performed a large-scale analysis of two mitochondrial (*CO1* and *CytB*) and one nuclear (*Myc*) DNA loci of *M*. *milvus*, *M*. *migr*. *migrans*, and their hybrids. In agreement with previous studies, we found low heterozygosity in *M*. *milvus* irrespective of the locus analyzed. We found that populations of both examined species were characterized by high gene flow within populations, which is not surprising given that kites are highly mobile animals [51]. All major haplotypes were distributed across the entire examined area, and only some of them displayed statistically significant aggregation in one region over others (Figs 1 and 4).

Summarized observational evidence from previous reports (Table 1) suggests incomplete reproductive isolation of *M*. *milvus* and *M*. *migr*. *migrans*. However, we found that Haldane´s Rule applies to sympatric populations of kites in central Europe. as there was no evidence for the presence of mitochondrial DNA of one species in individuals with the plumage of the other species except in F1 generation hybrids. Kites represent a relatively rare example of a zone of overlap and hybridization among Accipitriform raptors [6]. This means that there is a broad zone where the two species breed sympatrically but hybridize regularly, although at low frequency. Nests of hybrid pairs were observed most frequently in Germany, but also across the rest of the overlapping nesting range except Iberian peninsula, namely in Sweden, Czech Republic, Slovakia, Belarus, United Kingdom and Italy (Table 1). Such hybrid zones are thought to represent secondary contact after prolonged, or repeated, periods of allopatric speciation. We speculate that the existence of such zone in central Europe was facilitated by glacial refugia [55, 56] and, in the case of kites, by the Neolithic agricultural revolution [51, 57] as well as the subsequent expansion of farmers to areas with poor soil quality and/or higher altitudes, de/a-forestation events associated with the expansion and retreat of farmers from such sites, and hunting [58, 59]. Hunting, in particular, may play an important role in establishing a recent bottleneck in *M*. *milvus* populations. The persecution by humans in the past, especially at the beginning of the 20th century reduced the population size of this species, which was already not very diverse due to its evolutionary history [60]. Fragmentation of its range and isolation of its populations have led to strong declines in genetic variability. For example, in the Czech Republic, *M*. *milvus* was extinct for approximately one century when it re-colonized the country in 1974. Since then, it has increased in abundance up to the 125 breeding pairs recorded in 2015 [61, 62]. In the same country, *M*. *migr*. *migrans* has always been present, with the number of breeding pairs oscillating between 40–60 pairs in 2001–2003, 165–185 pairs in 2012, and 39 pairs in 2015 [61, 63]. Thus, a hybrid zone was formed, extending that found in Germany, as supported by both species sharing similar habitats throughout the study area (Fig 6). The sympatric coexistence of *M*. *milvus* and *M*. *migr*. *migrans* is documented back to the Neolithic period in Switzerland [64, 65] and to the Roman period in Germany [51, 64, 66–68]. Reintroduction programs have been implemented to support *M*. *milvus* in Scotland, Wales, the Balearic Islands, and southern Italy [50], which collectively have the potential to support the formation of more hybrid zones.

In conclusion, the central European population of *M*. *milvus* is clearly subject to free intraspecific gene flow. The meta-analysis of previously published data suggested that F1 and F2 hybrids are rarely observed to produce offspring with any kite species, and because we found that Haldane’s rule applies here, which leads to a complete absence of the offspring of hybrids among females involved in *M*. *milvus* or *M*. *migrans* nesting attempts throughout central Europe. Whether occasional gene flow occurs through the paternal line remains to be investigated, as the examined *Myc* gene displayed only marginal divergence between *M*. *milvus* and *M*. *migrans* despite GenBank data (sequences GU189490 and GU189491) suggesting strong interspecific differences in the examined *Myc* locus between *M*. *milvus* and *M*. *migr*. *migrans*. Further genomic research should elucidate, whether the suspected *Myc* hybridization occurred upon contacts prior to the hunting-driven temporary regional extinction of *M*. *milvus* throughout large part of its range or whether it is an artifact of the low genetic distance between the two kite species, which is one of the smallest among bird species [34, 51].

## Supporting Information

S1 Table Accession numbers of the consensus DNA sequences submitted to GenBank under accession numbers KU640396-KU640408 (*CO1* haplotypes), KU670077-KU670091 (*CytB* haplotypes) and KU708627-KU708835 (*Myc* sequences). (XLSX)
